# AlzStack: Forecasting early-onset Alzheimer's with an explainable AI system using multiple data balancing techniques^☆^

**DOI:** 10.1016/j.gloepi.2025.100235

**Published:** 2025-12-07

**Authors:** Venkata Aditi Modali, Manohar Pavanya, R. Vijaya Arjunan, D. Cenitta, Niranjana Sampathila, Radhika Kamath, Krishnaraj Chadaga

**Affiliations:** a Manipal Institute of Technology, Manipal Academy of Higher Education, Manipal, Karnataka, 576104, India; b Department of Obstetrics and Gynecology, Melaka Manipal Medical College (Manipal campus), Manipal Academy of Higher Education, Manipal, Karnataka 576104, India; c Department of Biomedical Engineering, Manipal Institute of Technology, Manipal Academy of Higher Education, Manipal, Karnataka 576104, India

**Keywords:** Alzheimer, Early diagnosis, XAI, Machine learning, Ensemble learning, Soft voting

## Abstract

Alzheimer's disease (AD) is a degenerative neurological disease that progresses over time, making early detection crucial for effective intervention and better patient prognosis. Traditional diagnostic methods such as cognitive assessments, neuroimaging, and biomarker analysis can be time-consuming, costly, and inconsistent. We introduce AlzStack, a soft voting ensemble model to classify AD from a richly detailed dataset containing 2149 patients across demographic, medical, lifestyle, and cognitive variables. To resolve class imbalance, we implemented a pipeline 5-fold cross-validation, randomized search for hyper parameter tuning and advanced resampling methods such as SMOTE (Synthetic Minority Oversampling Technique), ADASYN, BorderlineSMOTE, and SVMSMOTE. Soft Vote Classifier surpassed both stacking ensembles and hard voting with an AUC value of 94.27 %, accuracy of 93.26 %, precision of 89.17 %, a recall of 92.11 %, and F1-score value of 90.61 %.A secondary experiment with only resampling methods applied to data to all base models served as a baseline for comparison confirming the superior performance of cross-validation AlzStack configuration. To improve interpretability, we utilized a wide range of Explainable Artificial Intelligence (XAI methods) and these approaches yielded global and local explanations about model behavior, emphasizing key features like MMSE scores, functional measures, and behavioral markers. Combining robust predictive performance with explainable decision-making makes AlzStack is a healthcare decision-support algorithm for the early detection of AD.

## Introduction

**Fig. 1:**
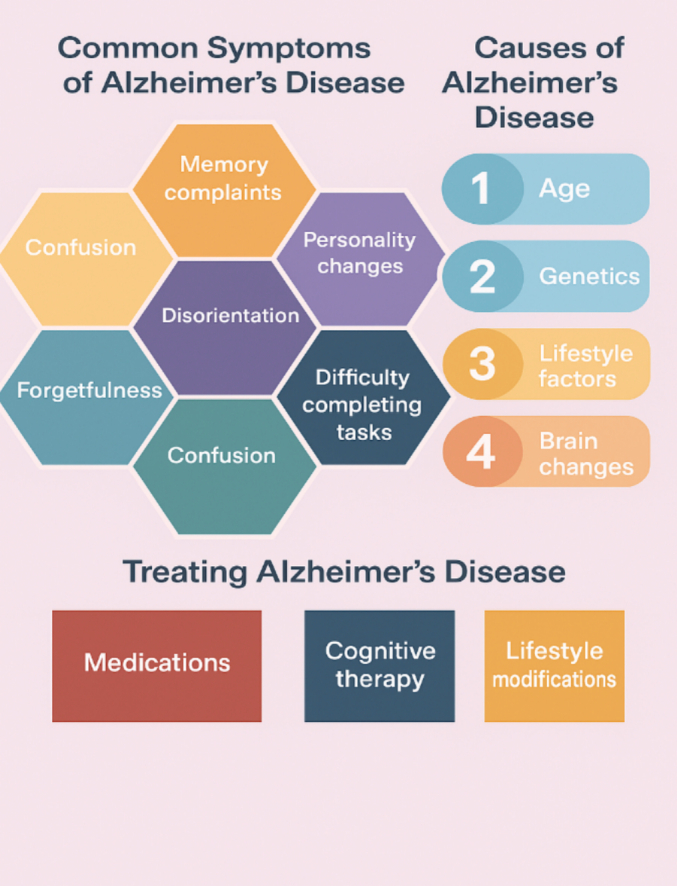
Key symptoms, causes, and management approaches for Alzheimer's.

Alzheimer's disease (AD) stands as the most prevalent neurodegenerative geriatric challenge. Its symptoms encompass dementia, decrease in cognition and behavior. According to the 2019 global burden of disease study, there were 28 million reported cases of AD in 2019, with estimates indicating that this number could rise to between 90 and 106 million by 2050 [1,2]. The complexity of diagnosing AD relies on a combination of clinical evaluation, clinical neuropsychological examinations, and cerebrospinal fluid (CSF) biomarker analysis, in combination with neuroimaging approaches such as magnetic resonance imaging (MRI) and positron emission tomography (PET) [3]. However, these traditional methods rely on significant levels of medical expertise, require considerable time, and often result in a delay in diagnosis. Early diagnosis of AD is needed to slow down neurodegenerative processes and support patient outcomes. Therefore, more efficient and accurate diagnosis methods are needed. Fig. 1 represents the key symptoms, causes, and management approaches for AD.

Machine learning (ML) is emerging as an effective measure of confirming the diagnosis of AD. It supports early diagnosis and intervention in AD through analyzing large-scale patient data, identifying subtle patterns, and improving diagnostic variability [4,5]. Various algorithms such as random forest (RF), XGBoost, and CatBoost have shown high degrees of predictive accuracy in diagnosing AD using large multimodal datasets (genetic, clinical, imaging data). Furthermore, oversampling methods such as Synthetic Minority Over-sampling Technique (SMOTE) and Adaptive Synthetic Sampling (ADASYN), have increased classifier performance by addressing the underlying class imbalance [6,7]. Medical experts can quickly and more consistently make diagnoses employing comprehensive ML models, decreasing the time to initiate early intervention and providing more personalized treatment plans.

The implementation of artificial intelligence (AI) in healthcare has transformed diagnosis and patient care. Most traditional AI models have produced encouraging results, but the “black box” nature of traditional AI context often limits clinician confidence to implement practically [8]. Explainable AI (XAI) offers a solution to this problem by explaining the reasoning behind the model's decision. The introduction of XAI frameworks alongside AI-assisted diagnostics will enable investigators to build trust and improve real world implementation [9].

Recent advances in ML and XAI have significantly impacted AD diagnostics, offering valuable contributions to both model performance and interpretability. El-Sappagh et al. proposed a two-layer RF model integrating 11 data modalities from 1048 ADNI dataset, reaching 93.95 % accuracy in diagnosis and 87.08 % in progression detection, enhanced by SHAP (SHapley Additive exPlanations) and rule-based explanations [10]. Kavitha et al. demonstrated that various machine learning models applied to the Open Access Series of Imaging Studies (OASIS) data showed that the RF and XGBoost models achieved higher accuracy. Additionally, the accuracy of the voting classifier model is also comparable to that of the RF and XGBoost models [11]. Table 1 describes a few studies on ML with AD.

**Table 1:** Description of studies on ML and AD.

Authors	Objective	Model used	Result	XAI used
1. Huh Y [12]	Ensemble learning-based AD classification	• ML classifiers: RF, XGB, and SVM • Ensemble model with soft voting	XGB accuracy: 0.864.	Nil
2. Lee A et al. [13]	Distinguishing patients with AD from health control through ML	RF	Accuracy: 97 % Recall:100 %	Nil
3. Uddin K et al. [14]	ML for early diagnosis of AD	RF, XGBoost, Voting Classifier, Gaussian NB, DT, and Gradientboost	Voting Classifier Accuracy 96 % Precision:100 % Recall: 46 %	Nil
4. Jiao B et al. [15]	ML for AD diagnosis using blood-based digital biomarkers	RF, SVM, KNN, LR (Logistic regression), Linear discriminant analysis and back propagation neural network.	RF Sensitivity 88.2 % Specificity 84.1 %	Nil
5. Ports K et al. [16]	ML to predict dementia	LR, LASSO, RF, and XGBoost.	High AUC values by LR: 0.83 LASSO:0.83 and XGBoost:0.82	Nil

Despite the growing body of research applying ML to AD prediction, several critical gaps remain.

First, a lot of current models ignore the incorporation of many data types, like demographic, lifestyle, and cognitive aspects, which can offer a more comprehensive knowledge of illness progression. Instead, they mostly rely on a small number of diagnostic modalities (such as imaging or biomarkers). Second, even though ensemble approaches have demonstrated potential for enhancing classification performance, the majority of research does not investigate or contrast various resampling techniques to handle the serious problem of class imbalance in AD datasets. While XAI methods are becoming popular, they are typically only used in single-tool approaches like SHAP, failing to take advantage of the advantages of numerous interpretability frameworks to obtain a deeper understanding of model behavior. Furthermore, it is common for classifier performance to be given without extensive optimization or assessment across a range of base learners. These restrictions impede the creation of reliable, comprehensible, and therapeutically useful AD diagnostic instruments.

This research has its design revolving around creating a diagnostic framework that is able to attain high predictive accuracy as well as bear resemblance with the diagnostic reasoning of neurologists. Due to the fact that the diagnosis of Alzheimer's disease is dependent on several factors, which includes cognitive, behavioral, and functional elements, it is our objective to build up a model that would be able to identify these interdependencies and, as a result, provide an explanation that is similar to the clinical judgment. Our underkying hypothesis is that combining ensemble method together with cross-validation, randomized hyperparameter tuning and an additional set of resampling strategies would lead to a model that would perform better in a more consistent way across folds and patient subgroups.Moreover, the combination of Explainable AI(XAI) methods such as SHAP, LIME, QLattice, and Anchor demonstrate how essential characteristics like MMSE, ADL, and functional assessment affect the forecasting of the model, thus improving both exactness and clarity. The main objective of this research is to devlop, validate, explain the new ensemble model, AlzStack, which is an effective for the early detection of Alzheimer's disease, by making clinically meaningful insights which ultimately increase it's applicability to real world clinical decision making.

By introducing AlzStack, a strong soft voting ensemble architecture for early AD classification, this study fills up the previously mentioned shortcomings. This study's main contributions are:

• ***Multidimensional Dataset Utilization:*** To provide a more comprehensive and representative foundation for AD prediction, AlzStack was constructed utilizing a comprehensive dataset of 2149 patients that contains demographic, lifestyle, medical, and cognitive factors.
• ***Systematic Handling of Class Imbalance:*** A comparative evaluation of advanced resampling techniques (SMOTE, ADASYN, BorderlineSMOTE, SVMSMOTE) was performed in conjunction with a wide range of classifiers (Support Vector Machine (SVM),K-Nearest Neighbor (KNN), Logistic Regression (LR), XGBoost, CatBoost, Random Forest (RF), AdaBoost) to improve model robustness.
• ***Robust Model Optimization* via *Cross-Validation and Randomized Search:*** This study uses a five-fold cross validation with randomized hyperparameter tuning across multiple resampling techniques to generalize performance and decrease bias caused by single train-test split.
• ***Superior Performance* via *Soft Voting:*** Through empirical evaluation, the soft voting ensemble (AlzStack) outperformed both hard voting and stacking methods, achieving high performance metrics (AUC: 94.27 %, Accuracy: 93.26 %, Precision: 89.17 %, Recall: 92.11 %, F1-score: 90.61 %), demonstrating its reliability in clinical prediction settings.
• ***Comparative Evaluation with Baseline framework:*** By conducting a parallel experiment with and without cross validation and randomized hyperparameter tuning,this study demonstrates cross validated AlzStack consistency across multiple validation folds and data resampling scenarios making it ideal for deployment in real world clinical environments.
• ***Comprehensive XAI Integration:*** Several explainable AI approaches, including SHAP, LIME, QLattice, and Anchor, were used to improve interpretability. These techniques offer a variety of viewpoints on feature significance and model behavior, which foster clinician trust and transparency.

## Materials and methods

### Dataset

**Fig. 2:**
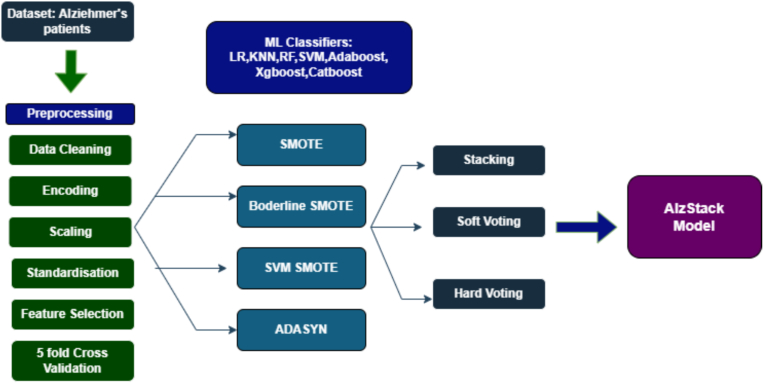
This figure represents the study procedure in building ALZSTACK.

The dataset was collected from a diverse group of individuals aged between 60 and 90 years of both genders, focusing on the diagnosis of AD. It is publicly available on Kaggle and the dataset therefore ethical clearance was not required. The dataset includes extensive patient information across 2149 records, encompassing demographic characteristics, lifestyle habits, medical history, clinical parameters, cognitive and functional assessments, and symptom profiles [17]. Thirty-two parameters were considered for building the model and presented in Table 2. Fig. 2 represents the study procedure.

**Table 2:** Markers used to predict Alzheimer's.

S·No	Feature	Description
1.	Age	60–90 years
2.	Gender	0: Male, 1: Female
3.	Ethnicity	0: Caucasian, 1: African American, 2: Asian, 3: Other
4.	Education level	0: None, 1: High School, 2: Bachelor's, 3: Higher
5.	Body Mass Index (kg/m^2^)	15–40
6.	Smoking	0: No, 1: Yes
7.	Alcohol consumption	Weekly alcohol consumption in units (0−20)
8.	Physical activity	Weekly physical activity in hours (0−10)
9.	Diet quality	Diet quality score (0–10)
10.	Sleep quality	Sleep quality score [[4], [5], [6], [7], [8], [9], [10]]
11.	Family history of AD	0: No, 1: Yes
12.	Cardiovascular disease	0: No, 1: Yes
13.	Diabetes	0: No, 1: Yes
14.	Depression	0: No, 1: Yes
15.	Head Injury	0: No, 1: Yes
16.	Hypertension	0: No, 1: Yes
17.	Systolic BP	Systolic Blood Pressure (90–180 mmHg)
18.	Diastolic BP	Diastolic Blood Pressure (60–120 mmHg)
19.	Cholesterol Total	Total cholesterol (150–300 mg/dL)
20.	Cholesterol LDL	LDL cholesterol (50–200 mg/dL)
21.	Cholesterol HDL	HDL cholesterol (20–100 mg/dL)
22.	Cholesterol Triglycerides	Triglycerides (50–400 mg/dL)
23.	Mini-Mental State Examination (MMSE)	0–30; lower = greater cognitive decline
24.	Functional assessment	Functional score (0–10); lower = more impairment
25.	Memory complaints	0: No, 1: Yes
26.	Behavioral problems	0: No, 1: Yes
27.	Activities of Daily Living (ADL)	0–10; lower = more impairment
28.	Confusion	0: No, 1: Yes
29.	Disorientation	0: No, 1: Yes
30.	Personality changes	0: No, 1: Yes
31.	Difficulty completing tasks	0: No, 1: Yes
32.	Forgetfulness	0: No, 1: Yes

### Statistical analysis

Table 3 describes the summary of study variables as mean, median, standard deviation(SD), interquartile range (IQR) and range.

**Table 3:** Summary statistics for several continuous attributes.

S·No	Variable	Diagnosis	N	Missing	Mean	Median	SD	IQR	Range
1.	Age	No	1389	0	74.95	75	8.9	16	30
		Yes	760	0	74.84	75	9.15	16	30
2.	BMI	No	1389	0	27.52	27.56	7.17	12.05	24.98
		Yes	760	0	27.91	28	7.3	12.76	24.97
3.	Alcohol consumption	No	1389	0	10.07	9.97	5.75	9.78	19.99
		Yes	760	0	9.98	9.86	5.77	10.39	19.92
4.	Physical activity	No	1389	0	4.91	4.73	2.87	4.93	9.98
		Yes	760	0	4.94	4.85	2.84	4.74	9.97
5.	Diet quality	No	1389	0	4.97	5.07	2.91	5.17	9.99
		Yes	760	0	5.03	5.08	2.91	4.98	9.95
6.	Systolic BP	No	1389	0	134.56	135	25.95	46	89
		Yes	760	0	133.72	133	25.96	44	89
7.	Diastolic BP	No	1389	0	89.78	90	17.67	30	59
		Yes	760	0	89.97	91	17.46	31	59
8.	Cholesterol total	No	1389	0	225	224.45	42.2	70.36	149.9
		Yes	760	0	225.57	226.45	43.19	74.06	149.75
9.	Cholesterol LDL	No	1389	0	125.36	124.84	43.42	74.72	149.58
		Yes	760	0	122.46	121.8	43.23	74.85	149.54
10.	Cholesterol HDL	No	1389	0	58.73	58.3	23.06	39.24	79.98
		Yes	760	0	60.8	61.85	23.24	40.88	79.94
11.	Cholesterol triglycerides	No	1389	0	226.57	226.14	101.91	176.07	349.53
		Yes	760	0	231.41	239.62	102.12	176.1	349.39
12.	MMSE	No	1389	0	16.27	17.15	8.93	16.05	29.99
		Yes	760	0	11.99	11.57	7.23	12.11	29.91
13.	Functional assessment	No	1389	0	5.86	6.24	2.76	4.27	9.98
		Yes	760	0	3.65	3.3	2.57	3.34	9.93
14.	ADL	No	1389	0	5.71	6.14	2.83	4.6	9.99
		Yes	760	0	3.66	3.24	2.7	3.6	9.94

**Fig. 3:**
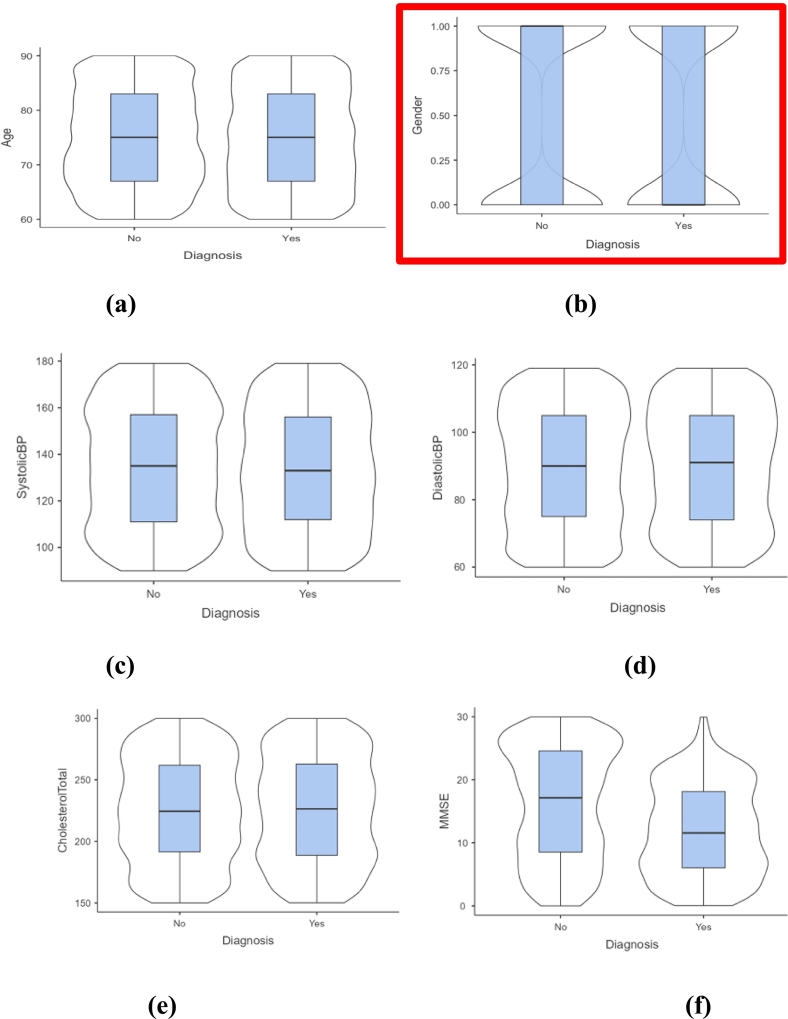
Violin plots depicting the distributional variation across selected markers. (a) Age (b) gender (c) SystolicBP (d) DiastolicBP(e), Cholesterol Total (f) MMSE.

Violin plots in Fig. 3 are used to visualize distributional differences via embedded boxplots. Violin plots of specific clinical characteristics categorized by AD are shown in Fig. 3. Although the distributions overlap somewhat, those with AD are often slightly older, which is consistent with age being a known risk factor. There is no discernible gender-based connection in this dataset, as gender seems to be balanced across both groups. Systolic and diastolic blood pressure measures exhibit comparable patterns across groups, indicating low predictive utility in the absence of further modification. Cholesterol levels are marginally higher in the AD group, though the overlap remains large. Notably, MMSE scores are significantly lower in the AD group, reflecting its utility as a cognitive assessment tool for detecting Alzheimer's-related decline.

### Data preprocessing

**Fig. 4:**
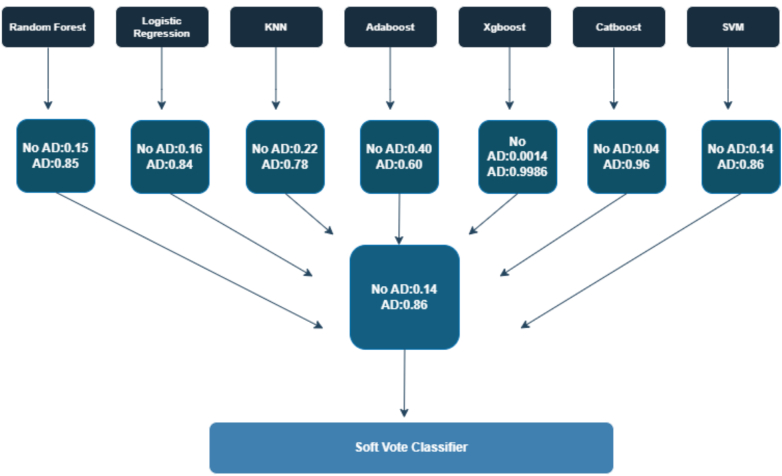
Overview of the AlzStack model architecture, constructed using seven baseline classifiers.

Data preprocessing is an initial step in ML that involves transforming and encoding raw data into an appropriate a format and facilitate building the prediction model. This process ensures that the data can be efficiently handled and interpreted by ML classifiers. This is particularly critical in supervised learning, as it potentially impacts the performance of the model. The volume of training data often increases exponentially with the dimensionality of the input space, making preprocessing indispensable. Proper data preprocessing mitigates overfitting and underfitting risks. Fig. 4 represents the ML pipeline in AD model.

**Data cleaning** involves finding and fixing or removing incorrect, incomplete, or noisy data. This step is crucial to enhance data quality and reliability. It typically addresses missing, irrelevant, or inconsistent records that could impair model performance.

**Normalization** is necessary when features have varying scales. Features with different magnitudes can bias the learning algorithm, leading to suboptimal performance. By scaling features to a common range, normalization ensures that all attributes contribute equally to the training. When features exhibit disparate numerical ranges, normalization adjust these features to a standard scale, thereby preventing outliers impacting the learning process [18].

**Missing data** is a common challenge, and its handling can significantly influence model's accuracy. Discarding entire rows with missing values often results in the loss of potentially valuable data, even if that data is incomplete. To maximize the information obtained from a dataset, missing values can be addressed through imputation. Each imputation algorithm employs a specific method to estimate these missing values; however, this does not guarantee that the estimated value is completely accurate. As a result, imputation can introduce noise and bias into the dataset, which is why it should be applied with caution [19].

**Hyperparameter tuning** is the process of choosing the effective set of hyperparameters that control the learning behavior of a model. Hyperparameters are set prior to training and significantly influence the learning process. Proper tuning ensures a good balance between generalization and overfitting. Techniques such as cross-validation and grid search are used in the current study. Cross-validation partitions the dataset into multiple folds, using each fold as a validation set while training on the remaining data. This helps identify issues such as overfitting or underfitting that may arise from data preprocessing errors. Grid search is considered an exhaustive and independent search method, testing all parameter combinations within a specified range to identify the best-performing model configuration. Despite its computational cost, it remains one of the most reliable tuning strategies when the search space is manageable. [[20], [21], [22]].

### Cross validation, grid search and resampling strategies

Model development and evaluation used a five fold stratified cross validation setup. This helped usa ensure model generalization and minimize bias due to train test split. Stratification kept the class balance the same in each fold. In every round, four folds went to training and one to validation. For each classifier, we figured out the mean plus or minus standard deviation of AUC, accuracy, sensitivity, and specificity. That way, we could check how stable and repeatable the models were across the folds. Hyperparameters got optimized with RandomizedSearchCV for each algorithm. It samples from the parameter options instead of checking every single combination like grid search does. This approach gives us about the same accuracy, but it saves a lot of computing time. Nested cross validation was used so that hyperparameter tuning within inner folds stayed separate from outer validation folds preventing optimistic bias.

All the preprocessing steps were inside an ImbPipeline from imbalanced learn. That included imputation, normalization, and fixing class imbalance. Missing values filled in with the median method. Continuous variables got standardized to zero mean and unit variance. Putting everything in the pipeline meant transformations only fitted on training data per fold. No data leakage happened, and evaluations stayed unbiased. Class imbalance between Alzheimer positive and control cases needed handling. We tested four oversampling methods in detail. Those were SMOTE, Borderline SMOTE, SVMSMOTE, and ADASYN. Each one came after scaling but before training in the pipeline. Synthetic samples for the minority class only came from training folds. We ran every resampling approach on all seven classifiers separately. The classifiers included CatBoost, XGBoost, Random Forest, AdaBoost, SVM, Logistic Regression, and KNN. This let us find the best matches between models and balancers.

Table 4 shows the full five fold cross validated results for all classifiers with each resampling. Borderline SMOTE and SVMSMOTE stood out for balanced performance. Their AUC scores stayed above 0.95 consistently, especially for gradient boosting like CatBoost and XGBoost. After tuning hyperparameters, the best resampling technique for each model resampling pair was used as input models for ensembles. Table 5 sums up how soft, hard, and stacking ensembles compared in performance. Table 6 puts baseline single split results next to cross validated ones. It shows steady improvements from combining resampling and validation.The whole setup with cross validation, randomized tuning, and resampling made for a solid evaluation process. It was fair, thorough, and easy to repeat. This built the base for the final AlzStack ensemble.

**Table 4:**
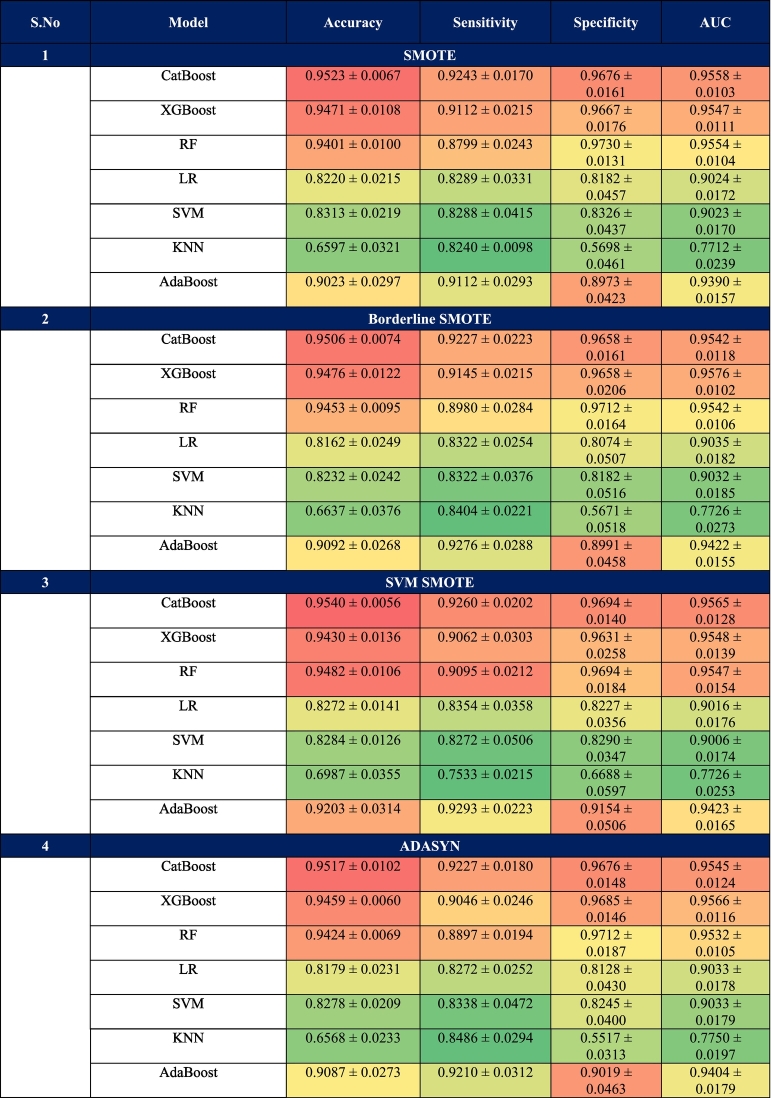
Different resampling techniques used in the study.

**Table 5:** Results of all ensemble methods.

Ensemble Method	AUC (Mean ± SD)	Accuracy (%)	Precision (%)	Sensitivity (Recall %)	F1-Score (%)
**Soft Voting (AlzStack)**	**0.9427 ± 0.0120**	**93.26 ± 1.15**	**89.17 ± 1.30**	**92.11 ± 1.80**	**90.61 ± 1.25**
Stacking Ensemble	0.9459 ± 0.0110	93.02 ± 1.30	88.61 ± 1.50	92.11 ± 1.90	90.32 ± 1.40
Hard Voting Ensemble	0.9306 ± 0.0130	92.56 ± 1.40	87.04 ± 1.60	92.76 ± 2.00	89.81 ± 1.55

**Table 6:** Baseline vs Cross Validated model Performance.

Model	Best Resampler (CV)	Validation Type	AUC (Mean ± SD)	Accuracy (%)	Sensitivity (%)	Specificity (%)	Δ AUC (Improvement)
Logistic Regression	Borderline SMOTE	Baseline / 5-fold CV	0.8697 → **0.9035 ± 0.018**	77.7 → **81.6 ± 2.5**	78.9 → **83.2 ± 2.5**	71.4 → **80.7 ± 5.1**	**+0.034**
KNN	ADASYN	Baseline / 5-fold CV	0.5489 → **0.7750 ± 0.020**	65.5 → **65.7 ± 2.3**	84.9 → **84.9 ± 2.9**	55.2 → **55.2 ± 3.1**	**+0.226**
SVM	ADASYN	Baseline / 5-fold CV	0.6424 → **0.9033 ± 0.018**	56.7 → **82.8 ± 2.1**	75.7 → **83.4 ± 4.7**	59.8 → **82.5 ± 4.0**	**+0.261**
Random Forest	SMOTE	Baseline / 5-fold CV	0.9379 → **0.9554 ± 0.010**	91.9 → **94.0 ± 1.0**	86.2 → **88.0 ± 2.4**	93.8 → **97.3 ± 1.3**	**+0.018**
AdaBoost	SVMSMOTE	Baseline / 5-fold CV	0.9350 → **0.9423 ± 0.017**	90.9 → **92.0 ± 3.1**	92.1 → **92.9 ± 2.2**	90.2 → **91.5 ± 5.1**	**+0.007**
XGBoost	Borderline SMOTE	Baseline / 5-fold CV	0.9419 → **0.9576 ± 0.010**	93.5 → **94.8 ± 1.2**	90.8 → **91.5 ± 2.2**	94.5 → **96.6 ± 2.1**	**+0.016**
CatBoost	SVMSMOTE	Baseline / 5-fold CV	0.9434 → **0.9565 ± 0.013**	94.9 → **95.4 ± 0.6**	92.8 → **92.6 ± 2.0**	94.3 → **96.9 ± 1.4**	**+0.013**

### Customized ensemble methods

This study looked into three ensemble learning strategies to boost classification precision, stability, and interpretability. These included Hard Voting, Soft Voting, and Stacking classifiers. Each one pulls together outputs from several base classifiers. This helps cut down on weaknesses from single models. It also lifts the overall predictive performance. The main setup in this work is called AlzStack. It builds on the Soft Voting Ensemble method. Here, seven tuned base classifiers got trained on their own. They are CatBoost, XGBoost, Random Forest, AdaBoost, SVM, Logistic Regression, and KNN. Every model gave a probability-based prediction for Alzheimer's-positive cases, marked as AD, and non-Alzheimer's cases, marked as No AD. Then those probabilities got averaged across the group. The final class came from the highest average probability among the classifiers.

Soft voting differs from Hard Voting in a key way. Hard Voting just goes by majority labels from the classes. Soft voting brings in the confidence from each model, through its predicted probabilities. This leads to decisions that feel more detailed and trustworthy. The way it mixes probabilities lets AlzStack strike a good balance between sensitivity and specificity. It keeps things interpretable too. That is a key part for helping with clinical choices. Fig. 4 shows the inside workings of the AlzStack ensemble. Each base classifier puts out probabilistic results. Those get merged in the soft-voting step to form the final prediction agreement. The design makes it better at handling new data it has not seen. It stays efficient on computing power and clear to follow.

The Stacking Ensemble, for its part, used a meta-learner based on logistic regression. That one learned from the probability outputs of the same base classifiers. The Hard Voting Ensemble, on the other hand, decided things just by majority vote. Table 5 lays out how they compared in performance. It shows the soft voting ensemble, which is AlzStack, hit the steadiest balance on every metric checked. Table 6 covers the gains from this ensemble setup over the basic models and the cross-checked single ones. All that backs up how solid the approach really is.

### XAI techniques

To promote interpretability and transparency this study utilizes a four cutting-edge XAI [23]. These were specifically selected to give holistic explanations to the model's decision at both global and local levels of predictions, allowing clinicians and scientists to gain a better understanding of how certain features like those from MMSE scores and other cognitive tests influence it. A brief description of XAI techniques is as follows.

**SHAP:** SHAP gives each attribute a value of importance that reflects how much it contributed to arriving at the prediction. SHAP in this work was utilized to provide global summary plots as well as beeswarm plots ranking attribute importance by its aggregate influence to model predictions within the data. These provide the key variables associated with Alzheimer's pathology [24,25].

**LIME:** interprets single predictions by perturbing local input data and training a straightforward interpretable proxy model to model the complex model's behavior near this instance [23,26]. This strategy was implemented to produce patient-level explanations, enabling clinicians to see why a model classified a specific patient as having or not having AD.

**QLattice** uses symbolic regression methods to identify explicit mathematical formulas describing relationships between features and model predictions. Through identification of compact and interpretable expressions, QLattice identifies nonlinear relationships between biomarkers and cognitive measures and reveals hidden patterns related to disease development [27].

**Anchor:** Anchor generates high-precision if-then rules which are “anchors” for certain model predictions. The rule-based explanations identify the circumstances where certain classifications are reliable and consistent, putting a more intuitive level of transparency into the model's decisions [9,28].

Comprehensive findings of XAI on AD is presented in the result and discussion section.

## Results

Four resampling methods were utilized in this work to handle imbalanced data, namely SMOTE, BorderlineSMOTE, SVMSMOTE, and ADASYN. The resampled data were trained using seven models, i.e., SVM, LR, KNN, XGBoost, CatBoost, RF and AdaBoost. As discussed in the earlier section, the proposed AlzStack algorithm is a Soft Voting Classifier, which yielded superior performance in terms of all evaluation metrics seen in Table 5.

To identify the best variant of each of the seven models trained for use within training AlzStack, all twenty-eight model-balancer combinations were assessed across five evaluation metrics namely accuracy, precision, recall, F1 Score, and AUC. The best variant of each model by AUC score was chosen as the representative one to be further utilized in training within the ensemble approach as outlined in Tables 4, CatBoost with SVM-SMOTE had an AUC score of 94.32 %, accompanied by Accuracy of 95 %, Precision of 93 %, Recall of 93 %, and F1 Score of 93 %, being the best-performing CatBoost configuration. Among all configurations, CatBoost-SVM-SMOTE achieved the best overall performance, with an accuracy of 0.95, precision of 0.93, recall of 0.93, F1 Score of 0.93, and AUC of 0.94. CatBoost-ADASYN and CatBoost-SMOTE, both showing accuracy of 0.94, F1 Scores of 0.92, and AUC of 0.94, demonstrating that CatBoost consistently performs well regardless of the resampling method used. In contrast, AdaBoost-SVM-SMOTE, while achieving a respectable recall of 0.91, had the lowest precision (0.84) among all models, resulting in a low F1 Score of 0.88, indicating a higher rate of false positives. RF-SVM-SMOTE reached an accuracy of 0.94 and precision of 0.93, but its recall dropped to 0.89, resulting in an F1 Score of 0.91. XGBoost-B-SMOTE and XGBoost-SVM-SMOTE, both of which maintained consistent F1 Scores of 0.91, albeit with slightly lower precision (0.91) and recall (0.91) than the top-performing CatBoost variants. Despite using different resampling strategies, most models maintained a high AUC of 0.94, indicating robust discriminative capability across classifiers. LR and SVM generally showed lower performance compared to ensemble methods. KNN had the weakest performance across all techniques, with accuracy around 0.50–0.52.

**Fig. 5:**
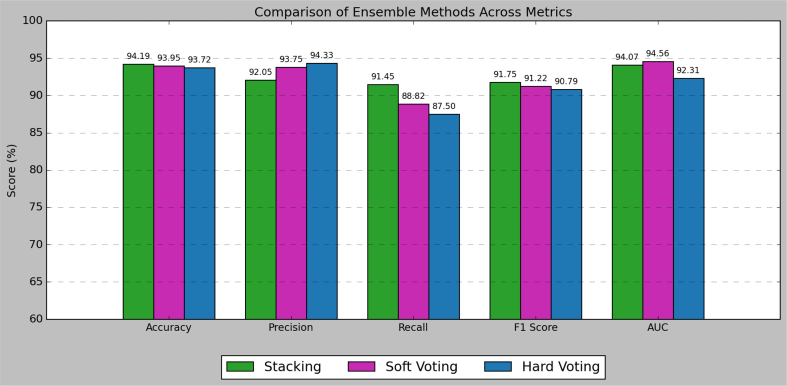
represents different metrics using stacking, soft and hard voting.

**Fig. 6:**
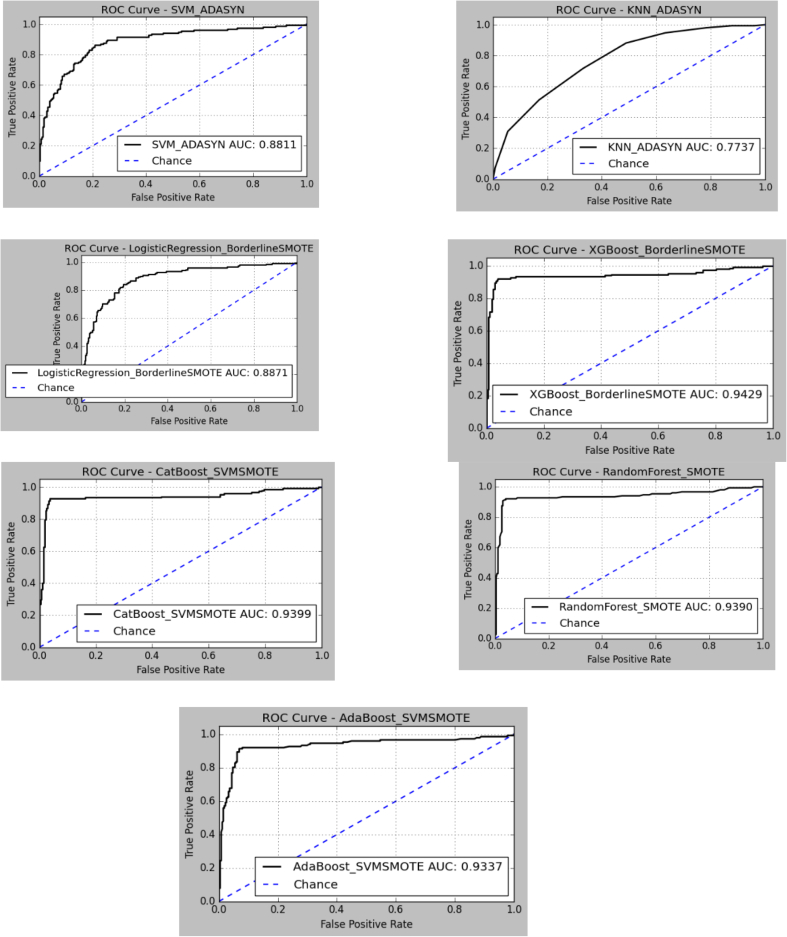
AUC curves for AlzStack model. (i) SVM (ii)KNN (iii) Logistic Regression(iv) XGBoost (v) CatBoost (vi) RF (vii) AdaBoost.

Table 5 represents the stacking and different voting methods used in the study. Fig. 6 represents graphical representation of the data. We applied each of the three ensemble strategies hard voting, soft voting, and stacking by blending predictions from the top-performing variants of basic models, namely, SVM, KNN, Logistic Regression, XGBoost, CatBoost, RF, and AdaBoost. All ensembles were designed to provide improved robustness, variance reduction, and generalizability. Through extensive cross-validation and performance assessment across several evaluation metrics such as accuracy, precision, recall, F1-score, and AUC, our best-performing model was found to be soft voting classifier-AlzStack consistently outperforming other ensemble strategies. This performance gain can be explained by effective averaging of probability scores, resulting in a better representation of confidence levels from each individual classifier. AlzStack's capacity for predictive accuracy while retaining interpretability makes it well-suited for clinical applications, where interpretable decision-supporting tools are crucial for facilitating early detection of AD. Fig. 5 represents the diagrammatic representation of different metrics.

These best-performing variants of each base model were thus combined to build the final Soft Voting Classifier so that each component contributed its best variant. The overall discriminatory power of the chosen models can also be seen in Fig. 6, where we have plotted the ROC-AUC for each model-balancer pairing, graphically affirming the better-than-expected choices made in the ensemble. These chosen variants corresponding to each algorithm's best-performing setup were subsequently combined in the final Soft Voting ensemble to provide a collective decision-making strategy optimized for best performance.

**Fig. 7:**
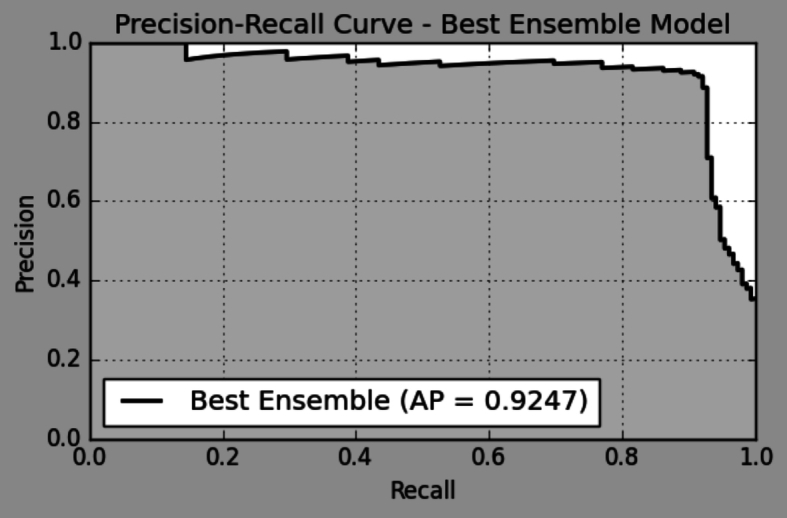
Precision-Recall curve for AlzStackApp (Soft Vote).

A further assessment with a Precision-Recall Curve, presented in Fig. 7, confirmed the model's impressive Average Precision (AP) score of 0.92, emphasizing its ability to preserve precision at even very high recall values.

**Fig. 8:**
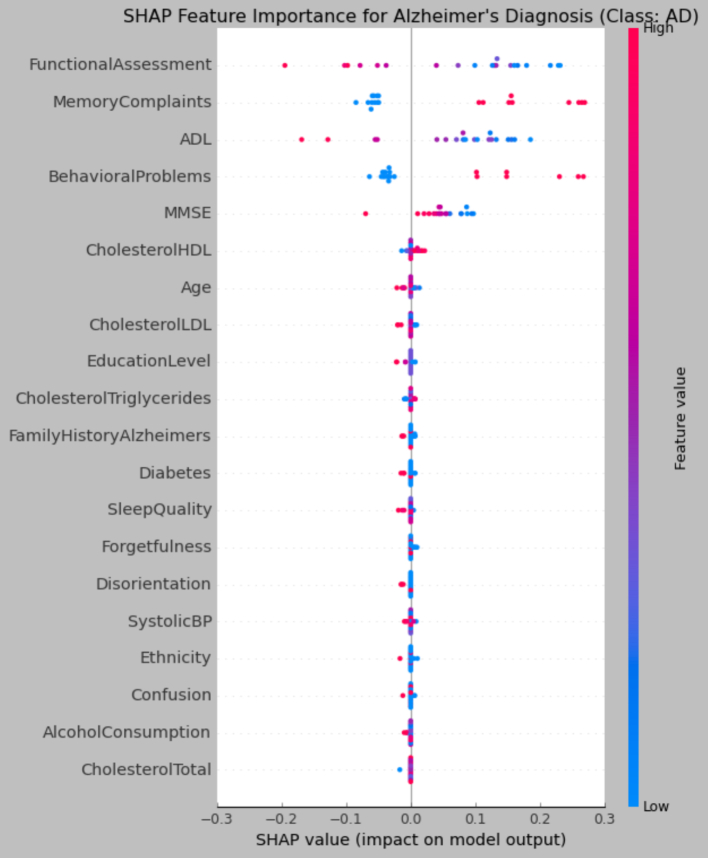
SHAP beeswarm plot illustrating feature contributions to Alzheimer's prediction.

To understand model predictions, SHAP were utilized, depicted in Fig. 8. This global interpretation scheme identified functional assessment, ADL, MMSE, memory complaints, and behavioral problems as the most significant predictors. The predictors were linked to early predictions of Alzheimer's. These findings are consistent with clinical knowledge and confer additional validity to model decision-making.

**Fig. 9:**
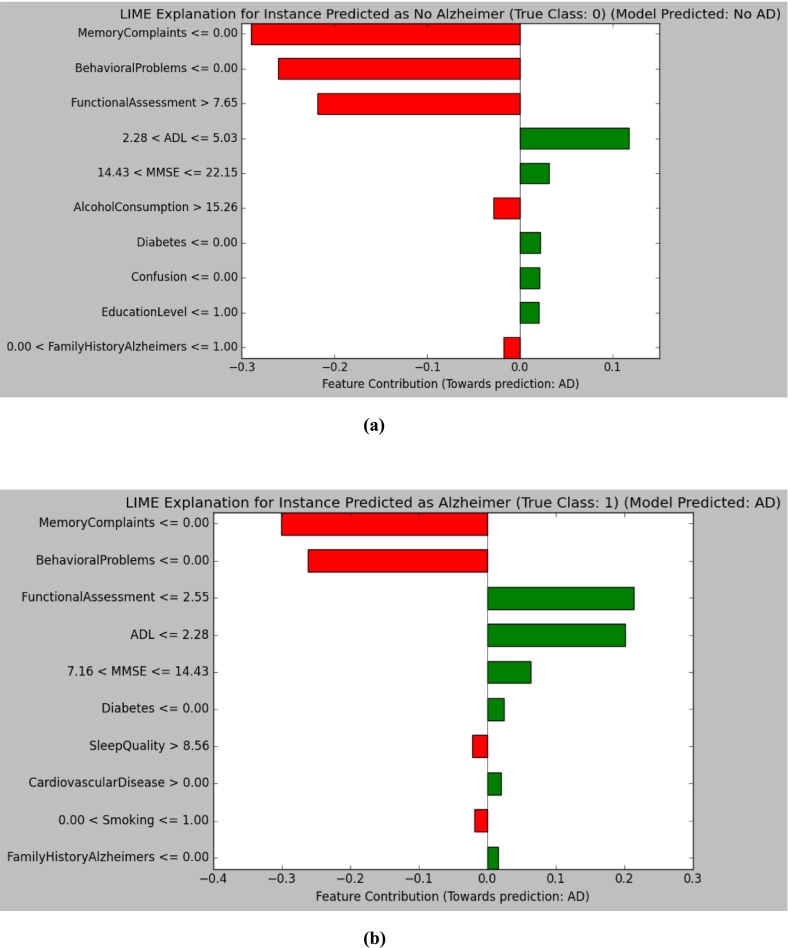
LIME model explanations: (a) Alzheimer's negative case, (b) Alzheimer's positive case.

To provide global explanations with local reasoning, LIME was applied to explain each instance's predictions, presented in Figs. 9. For a No Alzheimer's diagnosis, not having memory complaints and behavioral problems, along with a large functional assessment value, were major contributors. For Alzheimer's, memory complaints along with low Functional assessment scores and ADL, with behavioral problems, were major contributors to this prediction. These local instance-level insights not only verify model outputs but provide clinicians with interpretability.

**Fig. 10:**
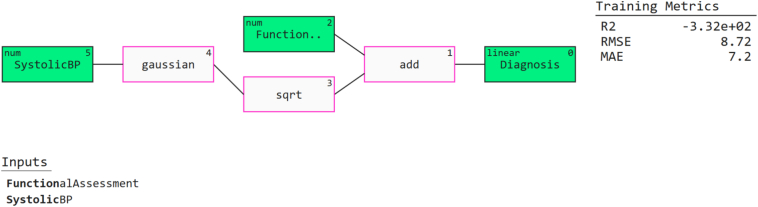
Q-Graphs to interpret important markers in Alzheimer's prediction.

Fig. 10 represents the Q-graph. To investigate symbolic and compact models of the diagnostic process, a QLattice model was trained with just two features Functional Assessment and Systollic Blood Pressure presented in Fig. 10. The two features went through a Gaussian node and then into a linear output node for predicting AD. The QLattice model was extremely interpretable yet verified the importance of these two features. Its performance was low, with MAE at 7.2 and R^2^ at −0.0332, RMSE at 8.72. This indicates QLattice is best for transparency but not predictive capabilities like ensemble models. The identification of Functional Assessment and Systollic Blood Pressure as significant contributors corroborates earlier findings from SHAP and LIME, instilling confidence in them.

Besides feature attribution techniques, Anchor generated rule-based explanations with high-precision rules, interpretable by humans. As presented in Table 7, one rule labeled Alzheimer's with 100 % precision was: Functional Assessment ≤2.55, ADL ≤ 5.03, MMSE ≤22.15, whereas another rule labeled No Alzheimer's with 95 % precision was: Functional assessment >7.65, memory complaints ≤ 0. The rule-based anchors align with domain expertise and provide strong clinical decision-support diagnostic cues.

**Table 7:** Model interpretability using Anchor to explain Alzheimer's diagnostic decisions.

Instance	Predicted Class	Anchor Conditions	Precision	Coverage
0	AD	Functional Assessment ≤2.55 ADL ≤ 5.03 MMSE ≤22.15	1	0.11
1	No-AD	Functional Assessment >7.65 Memory Complaints ≤ 0.00	0.95	0.19

Table 8 represents the comprehesive results for AD with XAI methods. ADL and functional assessments score are chosen as important predictors by 3 XAI methods. MMSE was chosen by 2 XAI methods. Systolic Blood Pressure, behavioral problems, memory complaints are also chosen as influential.

**Table 8:** Comprehensive predictors by XAI techniques.

SHAP	LIME	QLATTICE	ANCHOR
• Functional Assessment • ADL • MMSE • Behavioral Problem A	• Memory Complaints • Functional Assessment • ADL • Behavioral Problem	• Functional Assessment • Systolic Blood Pressure Functional Assessment • ADL • MMSE	• Functional Assessment • ADL • MMSE

## Discussion

Our work adds to this growing landscape by introducing AlzStack, a soft voting ensemble model trained with a rich, heterogeneous dataset containing demographic, clinical, lifestyle, and cognitive variables for 2149 patients. By using a large variety of base models SVM, KNN, LR, XGBoost, CatBoost, RF, and AdaBoost and resampling methods such as SMOTE, ADASYN, BorderlineSMOTE, and SVMSMOTE, we effectively countered the clinical data's inherent imbalanced class.

The ensemble strategy, specifically soft voting trained on based models evaluated under fivefold cross validation, showed better performance, with AUC of 94.27 and accuracy of 93.26 %. Compared to baseline models cross validated models consistently performed better confirming the stability of integrated resampling and cross validation strategy as observed in Table 6. Significantly, AlzStack also leveraged multiple XAI methods SHAP, LIME, QLattice, and Anchor to analyze feature importance and model behavior, enabling transparent revelation of the model's decision-making pathway. These interpretive tools identified notable contributors to AD risk, enabling clinical decision-making and promoting trust in AI diagnostics. Table 9 summarizes key recent studies that utilize various XAI.

**Table 9:** different studies which involved XAI techniques in AD diagnosis.

Reference	Objective	Algorithms	XAI
1. Junior K [24]	Multiclassification of AD	Deep learning models-RESNET	LIME,GRAD-cam
2. Bhattarai P et al. [29]	XAI in neuroimaging biomarkers in AD	deep learning neural network	SHAP
3. Cai J et al. [30]	Prediction of conversion from mild cognitive impairment (MCI) to AD	Explainable Boosting Machine (EBM)	Global explanation, local interpretation
4. Mahmud T et al. [31]	AD diagnosis using deep transfer learning	VGGNets (very deep convolutional networks) and DenseNets models	Saliency maps and grad-CAM
5. García-Gutiérrez, Fernando et al. [32]	variations in brain metabolism from MCI to dementia	DL models	Integrated gradient
6. Sarica A et al. [33]	Gender specific conversion from MCI to AD	Random Survival Forests (RSF)	SHAP
7. Bogdanovix B et al. [34]	In-depth insights into AD with huge dataset	XGBoost	SHAP
8. Leandrou, Stephanos et al. [35]	quantitative imaging in the assessment of AD	XGBoost	SHAP
9. Amoroso, Nicola et al. [36]	Brain connectivity in AD	Brain connectivity model	SHAP
10. Proposed study	ALZSTACK – Forecasting early onset of AD	SVM, KNN, Logistic Regression, XGBoost, CatBoost, RF, AdaBoost + ALZSTACK (Soft Voting Ensemble)	SHAP, LIME, QLattice, Anchor

Early diagnosis of AD, especially during the preclinical stage when cognitive symptoms have not yet appeared, is one of the major obstacles in AD. Effective intervention relies on the ability to diagnose early, but current diagnostic methods, such as CSF analysis and positron emission tomography (PET) scans,requires expertise, resources and time consuming [37]. Most of the research on diagnostic methods for AD. Cognitive function tests, activities of daily living (ADL) evaluations, and family history assessments all help determine whether a patient exhibits symptoms of dementia. Neurodegeneration occurs at the molecular level, and symptoms typically do not manifest until there has been a significant and permanent loss of neurons. Consequently, making an early diagnosis can be challenging. Currently, once symptoms appear, the available treatments primarily focus on reducing symptoms or delaying the progression of the condition. The main challenge in developing treatments for neurodegenerative diseases lies in diagnosing the early pathogenic mechanisms that lead to cell death and to protect the affected neurons [38].

ML and automated segmentation methods have shown substantial results in a variety of computer vision and image processing in AD images. Direct feature extraction from picture data is made possible by more modern ML techniques, especially those that use deep convolutional neural networks (CNNs), in a data-driven fashion [[39], [40], [41], [42], [43], [44], [45]]. Our results suggest ADL and functional assessments as important early predictors for forecasting AD. Therefore, monitoring ADL may help in the early diagnosis of AD. To fully reflect the clinical impact of AD, regulatory advice has underlined the significance of evaluating both cognitive and functional outcomes in AD trials [46].

MMSE has also been selected as one of the predictor variables. It is a brief neuropsychological test that provides an overview of cognition. In patients with Mild Cognitive Impairment (MCI), more specialized neuropsychological tests are employed to assess various domains such as language, praxis, and executive functions, in addition to the MMSE. Its benefits include ease of use, particularly concerning time and resources, as well as the absence of direct negative consequences. Furthermore, it enjoys high acceptance among medical professionals who treat dementia patients [47].

We also employed ensemble technique with multiple classifiers to build a robust model to forecast AD. The model has a high sensitivity and specificity in detection of AD.Sofy voting has high AUC showing the model's performance.

## Conclusion

This work presents AlzStack, a new soft voting ensemble designed specifically for accurate, early diagnosis of Alzheimer's from rich, multi-dimensioned data. The data includes demographic, clinical, cognitive, and lifestyle variables, reflective of AD's multifactorial nature. In a measure to address the imbalanced nature of medical data, we applied a five fold cross validation pipline integrating resampling methods SMOTE, ADASYN, BorderlineSMOTE, and SVMSMOTE along with seven base classifiers SVM, KNN, Logistic Regression, XGBoost, CatBoost, RF, and AdaBoost. Several limitations need to be mentioned despite promising findings. The retrospective nature and small sample size can affect generalizability. Future directions can include multimodality integration, prospective validation in heterogeneous cohorts, and deployment within federated learning frameworks for providing privacy and scalability. Briefly, AlzStack presents a clinically effective, interpretable AI solution for early Alzheimer's diagnosis. Its effective performance with multiple-level interpretability makes it a promising means for timely intervention, optimization of patient outcomes, and reducing the social impact of Alzheimer's. Furthermore, data security innovations such as steganography and cryptographic techniques can be applied to protect patient data integrity and confidentiality, to enable more extensive deployment of AI-based diagnosis systems. Continued research toward developing better XAI approaches will similarly be necessary to ensure interpretability to align closely with clinical reasoning, providing optimal clinical utility from AI models. Altogether, AlzStack represents a significant step toward applying interpretable machine learning models to Alzheimer's diagnosis. Its accuracy, paired with robust interpretability, makes it a useful tool in early detection of AD and clinical. Through overcoming current limitations, AlzStack and similarly designed models hold promise to fundamentally change AD diagnosis, improving healthcare.

## CRediT authorship contribution statement

**Venkata Aditi Modali:** Software, Writing – original draft. **R. Vijaya Arjunan:** Supervision, Project administration. **D. Cenitta:** Methodology, Conceptualization. **Niranjana Sampathila:** Supervision, Project administration. **Radhika Kamath:** Software, Investigation, Formal analysis. **Krishnaraj Chadaga:** Methodology, Data curation, Conceptualization. **Manohar Pavanya:** Writing – review & editing.

## Ethical approval

Not applicable.

## Funding

No funding was available for this research.

## Declaration of competing interest

The authors declare that they have no known competing financial interests or personal relationships that could have appeared to influence the work reported in this paper.

## Data Availability

Data will be made available by the authors on prior request.
